# Immunosensor Enhanced with Silver Nanocrystals for On-Chip Prostate-Specific Antigen Detection

**DOI:** 10.3390/bios15070428

**Published:** 2025-07-03

**Authors:** Timothy A. Okhai, Kefilwe V. Mokwebo, Marlon Oranzie, Usisipho Feleni, Lukas W. Snyman

**Affiliations:** 1 Clinical Engineering Group, Department of Electrical Engineering, Faculty of Engineering and the Built Environment, Tshwane University of Technology, Pretoria 0183, South Africa; 2 Department of Electrical Engineering, College of Science, Engineering and Technology, University of South Africa, Florida Campus, Johannesburg 1710, South Africa; snymalw@unisa.ac.za; 3 SensorLab, Department of Chemistry, University of the Western Cape, Cape Town 7535, South Africa; 3696304@myuwc.ac.za (K.V.M.); 3338704@myuwc.ac.za (M.O.); 4 Institute for Nanotechnology and Water Sustainability (iNanoWS), College of Science, Engineering and Technology, University of South Africa, Florida Campus, Johannesburg 1710, South Africa; felenu@unisa.ac.za

**Keywords:** biosensors, cancer, immunosensors, nanomaterials, on-chip, prostate-specific antigen, silver nanocrystals

## Abstract

An electrochemical immunosensor for the quantification of prostate-specific antigens (PSAs) using silver nanocrystals (AgNCs) is reported. The silver nanocrystals were synthesized using a conventional citrate reduction protocol. The silver nanocrystals were characterized using scanning electron microscopy (SEM) and field effect scanning electron microscopy (FESEM), X-ray diffraction (XRD), high-resolution transmission electron microscopy (HRTEM), Fourier-transform infrared spectroscopy (FTIR), UV-Vis spectroscopy, and small-angle X-ray scattering (SAXS). The proposed immunosensor was fabricated on a glassy carbon electrode (GCE), sequentially, by drop-coating AgNCs, the electro-deposition of EDC-NHS, the immobilization of anti-PSA antibody (Ab), and dropping of bovine serum albumin (BSA) to prevent non-specific binding sites. Each stage of the fabrication process was characterized by cyclic voltammetry (CV). Using square wave voltammetry (SWV), the proposed immunosensor displayed high sensitivity in detecting PSA over a concentration range of 1 to 10 ng/mL with a detection limit of 1.14 ng/mL and R^2^ of 0.99%. The immunosensor was selective in the presence of interfering substances like glucose, urea, L-cysteine, and alpha-methylacyl-CoA racemase (AMACR) and it showed good stability and repeatability. These results compare favourably with some previously reported results on similar or related technologies for PSA detection.

## 1. Introduction

The detection, monitoring and management of tumours in clinical medicine and oncology has been enhanced by recent advancements in the discovery of different tumour biomarkers. Among these biomarkers are alpha-fetoprotein (AFP), used to assess the baby’s risk of birth abnormalities and genetic conditions such as Down’s syndrome and neutral tube defect during pregnancy; cytokeratin 19 fragment (cyfra 21-1), a serum marker for detecting lung cancer, oesophageal cancer, head and neck cancer, anal canal cancer and gynaecological cancer, and cancer antigen-125 (CA125), an antigenic tumour marker that can diagnose or monitor ovarian, fallopian tube or primary peritoneal cancer. Others are carcinoembryonic antigen (CEA), a tumour biomarker linked with liver, colorectal, ovarian, breast and lung cancer, and prostate-specific antigen (PSA), the biomarker for detecting prostate cancer [1,2,3]. The most common cancer in males worldwide, which is prostate cancer, poses a significant health risk. Research indicates that the standard prostate-specific antigen (PSA) level in normal human serum is below 4 ng/mL; however, values over 20 ng/mL are frequently linked to the existence of prostate cancer [4,5,6,7,8]. Early detection of this disease is crucial for improving survival rates and treatment outcomes. This is typically accomplished by measuring PSA levels in the blood. The enzyme-linked immunosorbent assay (ELISA) is the standard method used in many clinical environments for PSA detection [9]. However, this approach has some drawbacks, including the setup cost, as it requires expensive laboratory equipment and skilled personnel, and a long processing time for the results, which can take several hours or days to process. Consequently, there is a pressing need for a detection method that addresses these limitations by offering a rapid, cost-effective, and user-friendly solution to enhance early detection and improve survival rates for prostate cancer patients.

Recent studies have explored various innovative detection tools for prostate-specific antigens to enhance the accuracy and specificity of prostate cancer diagnosis [10,11]. These advanced detection technologies include aptasensors, enzyme-based sensors, and immunosensors. Aptasensors utilize nucleic acid aptamers that bind specifically to PSA molecules, allowing for the detection of low concentrations of PSA in biological samples. They have high specificity and sensitivity and have been shown to achieve detection limits in the picomolar range. Their ease of use, rapid response time, and the possibility of integration into portable devices for point-of-care testing make them suitable for early diagnosis of prostate cancer [10]. Enzyme-based sensors typically involve immobilizing enzymes that catalyse reactions, producing measurable signals in the presence of PSA. For example, Oliveira et al. have explored using horseradish peroxidase (HRP) conjugate with anti-PSA antibodies to create a colorimetric assay that provides a visual indication of PSA levels [11]. This method is simple, cost-effective, and maintains adequate sensitivity for clinical applications. Immunosensors employ antibodies that specifically recognize PSA to provide quantitative measurements. They accomplish this by employing bovine serum albumin (BSA) to obstruct non-specific sites and concentrate exclusively on the specific sites of the antibodies, a process crucial for enhancing the specificity and accuracy of the sensor. When a sample containing PSA is introduced, the PSA molecules bind non-specifically to the capture antibodies. The blocked non-specific sites ensure that only PSA binds to the sensor, reducing background noise and improving the accuracy of the detection [12,13]. The change in the sensor’s signal is proportional to the concentration of PSA in the sample. This allows for the quantitative measurement of PSA levels, with very high sensitivity [13]. Recent advances in this field have focused on enhancing the sensitivity and selectivity of immunosensors by modifying them with various modifications, such as nanomaterials, to amplify the signals produced [14]. This work focuses on developing an electrochemical immunosensor that utilizes silver nanocrystals to significantly improve the detection limit. This facilitates PSA detection levels at concentrations much lower than traditional methods, potentially reducing false positives and unnecessary biopsies.

Silver nanoparticles significantly enhance the sensitivity of immunosensors through several mechanisms that leverage their unique chemical and physical properties. They have a high surface area relative to their volume, allowing for increased binding sites for target biomolecules [15]. Their signal amplification capabilities are due to their ability to dissolve in certain systems and to release silver ions (Ag+), which can then interact with fluorescent probes or other signalling molecules to significantly amplify the detectable signals [16]. They exhibit excellent electrocatalytic properties, which improve the electrochemical response of immunosensors [15]. Their ability to facilitate electron transfer enhances the sensitivity of electrochemical detection methods, allowing for lower limits of detection compared to conventional techniques. The unique optical properties of silver nanoparticles enable surface plasmon resonance, which enhances the optical signals in biosensing applications [15]. Furthermore, the inherent antimicrobial properties of silver nanoparticles help maintain sensor performance by reducing contamination risks during assays [16]. Finally, their versatility in functionalization ensures that silver nanoparticles can be easily functionalized with various biomolecules (e.g., antibodies), allowing for tailored sensor designs that enhance specificity and sensitivity for particular targets [15]. This is crucial for developing highly specific immunosensors capable of detecting a wide range of biomarkers. These advantages make silver nanocrystals a valuable component in developing advanced biosensing technologies for clinical diagnosis and other applications requiring high sensitivity and specificity. In our previous study on nanomaterial-enhanced receptor technology for silicon on-chip biosensing applications [17,18], we demonstrated the successful integration of both basic and advanced micro- and nanoscale sensors onto silicon substrates by leveraging silicon avalanche-mode light-emitting devices (Si-AMLEDs) in conjunction with standard silicon integrated-circuit fabrication techniques. These sensors could detect a range of physical and biochemical parameters through mechanisms involving waveguide optics, evanescent field interactions, and waveguide-integrated receptor layers. Furthermore, we proposed that the incorporation of nanomaterials within the receptor cavity could enhance the selectivity and sensitivity of the biosensing platform. Building upon this foundational work, the present study introduces a novel electrochemical immunosensor for the quantitative detection of prostate-specific antigen (PSA), employing silver nanocrystals (AgNCs) as signal amplification agents. This sensor is designed with future integration into a silicon photonic microchip in mind, enabling rapid, point-of-care diagnostic applications. The innovation of this work lies in the strategic use of AgNCs to enhance signal transduction within the receptor cavity, thereby facilitating their potential incorporation into Si-AMLED-based biosensing systems.

## 2. Materials and Methods

### 2.1. Chemicals and Equipment

For the synthesis of the silver nanocrystals, the chemicals used were silver nitrate (AgNO_3_ 99+%), trisodium citrate dihydrate (Na_3_C_6_H_5_O_7_), sodium borohydride (NaBH_4,_ 98%), sodium chloride (NaCl), sodium bromide (NaBr, 98%), potassium iodide (KI) and ascorbic acid (AA). All these chemicals were purchased from Sigma Aldrich, South Africa, and were used as received. The chemicals used to prepare the immunosensor were monoclonal anti-PSA antibody, EDC-NHS, and BSA, all purchased from Sigma Aldrich. The PSA is of analytical grade and was also purchased from Sigma Aldrich. All glassware was cleaned with aqua regia (3:1 *v*/*v* HCl (37%)/HNO_3_ (65%) solutions) and then rinsed thoroughly with H_2_O before use. All experiments utilized Milli-Q water (18 MΩ cm, Millipore). Various analytical methods and tools were used to characterize the AgNCs. The JSM-7800F Field Emission Scanning Electron Microscope (FESEM, JOEL Ltd., Akishima, Japan) was used to examine the surface structure and elemental signature of the silver nanocrystals, paired with energy-dispersive X-ray spectroscopy (EDS). The functional groups were studied using the KBr pellet technique on a Frontier PerkinElmer Fourier-transform infrared (FTIR) spectrometer (Spectrum 100 spectrometer, PerkinElmer, Waltham, MA, USA) in the 400–4000 cm^−1^ wavelength range. To determine the particle size distributions, a transmission electron microscope model JEOL JSM 100 CX II (JOEL Ltd., Akishima, Japan) was utilized, with the maximum voltage being set at 100 kV. GraphPad Prism^®^ Version 5.03 was used to plot the TEM images and results. The PANanalysis XRD equipment and High Score software (version HighScore Plus) were used for the X-ray diffraction (XRD) analysis. Using Origin 2021 software, all of the data gathered from the characterization apparatus were plotted.

### 2.2. Synthesis of Silver Nanocrystals

A slightly modified technique adapted from Li et al. [19] was used to synthesize the AgNCs. The following protocol was followed in the synthesis of the AgNCs: this citrate reduction procedure involved dissolving 9 mg of AgNO_3_ in 50 mL of H_2_O and bringing it to the boil while stirring. The boiling AgNO_3_ solution was then quickly mixed with 1 mL of a sodium citrate (1 wt %) aqueous solution, while being vigorously stirred. As the reaction time increased, the reaction solution’s colour altered from colourless to yellow to turbid and brown. To increase the production yield of AgNCs, the resulting brown solution was kept boiling, while being stirred for an hour. After cooling to room temperature, the solution was kept at 4 °C until it was needed.

### 2.3. Immunosensor Fabrication Process

**Figure 1:**
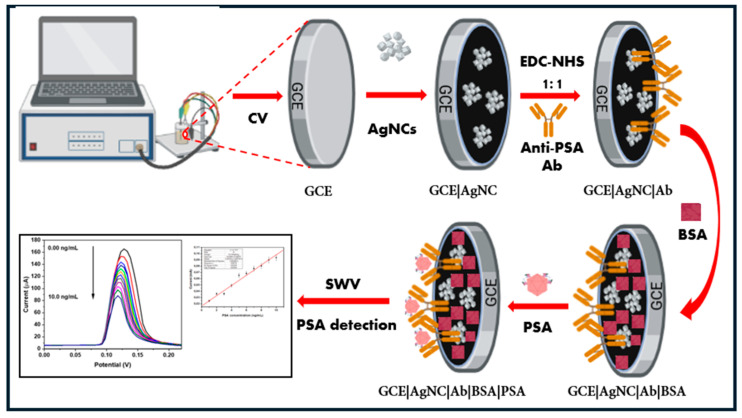
Schematic showing the immunosensor fabrication process.

Before being employed, the glassy carbon electrode (GCE) was meticulously polished to provide a smooth, consistent surface, and sonicated for 30 min. Each step of the immunosensor development process was characterized by cyclic voltammetry (CV). First, 5 mL of 10 mM phosphate buffer solution (PBS) of pH 7.4 was measured and poured into a glass vial with a three-electrode setup, i.e., the working electrode, the reference electrode, and a platinum wire counter electrode. Then, CV measurements were carried out on the bare GCE at a scan rate of 50 mV/s in the potential window of −1.0 to 1.0 V, and the measurement values were recorded for the bare GCE. Then, the GCE was modified with 10 μL (10 mg/mL) of AgNCs and left overnight to dry before the CV was repeated. This time, the measured values were labelled as GCE/AgNC. After gentle rinsing with deionized water and PBS, the electrode was modified with 1-ethyl-3-(3-dimethylaminopropyl) carbodiimide/N-hydroxy succinimide (EDC/NHS) cross-linking chemistries for 30 min. at room temperature, for activation of the amine groups to bind covalently to the carboxyl groups of the antibody. Next, 50 μL of (10 μg/mL) of the antibody (Ab) was drop-coated on the electrode and left to dry for 1 h, forming the GCE|AgNC|Ab. Next, 20 μL of 1% BSA was drop-coated onto the electrode for 1 h to block non-specific binding sites, forming the GCE|AgNC|Ab|BSA immunosensor. Each stage of the fabrication process was characterized by CV and SWV in 10 mM PBS (pH 7.4) at a scan rate of 50 mV/s in the potential window of −1.0 to 1.0 V. Finally, different concentrations of the PSA antigen were spiked into the electrochemical cell for detection, via SWV. The fabricated immunosensor GCE|AgNC|Ab|BSA was refrigerated at 4 °C when not in use. Figure 1 is the schematic representation of the immunosensor fabrication process.

### 2.4. Experimental Measurements

The experimental procedure involves electrochemical measurements performed on a three-electrode system in a glass vial. This consists of a platinum wire counter electrode, a reference electrode, and a 3 mm glassy carbon electrode (GCE), which serves as the working electrode. The experimental parameters for the CV measurements were an E-step of 5 mV, t-equilibration of 2 s, a current range of 1 mA, a scan rate of 50 mV/s, and a potential window of −1.0 to 1.0 V. The supporting electrolyte was 10 mM phosphate buffer solution (pH 7.4). A pulse duration of 10 ms, a pulse amplitude of 10 mV, a current of 1 µA, a scan rate of 50 mV/s, and an equilibration time of 5 s were the experimental settings for the differential pulse voltammetry (DPV). For the square wave voltammetry (SWV), the experimental parameters were a pulse amplitude of 25 mV, a current range of 1 µA, a scan rate of −1.0 to 1.0 V, an E-step of 5 mV, a frequency of 10 Hz, and a t-equilibration of 2 s.

## 3. Results and Discussion

### 3.1. Characterization of Nanomaterials

**Figure 2:**
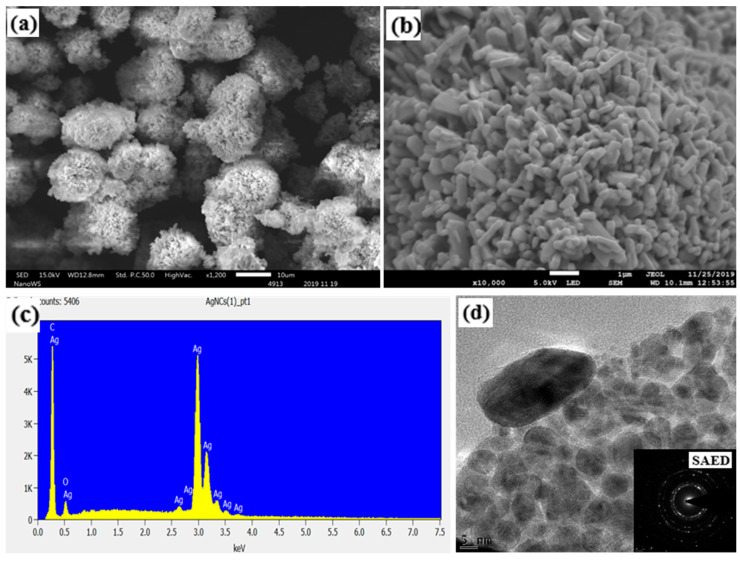
(**a**) SEM photomicrograph at ×1200, (**b**) FE-SEM photomicrograph at ×10,000, (**c**) energy dispersion X-ray spectroscopy graph, and (**d**) transmission electron microscopy image.

Figure 2a,b illustrate the scanning electron microscope (SEM) and field-effect scanning electron microscope (FESEM) topographical images of the nanoparticles. These equipment were used to investigate the shape, size, impurities, and stability of the particles. The globular-shaped morphology of the AgNCs shown in the SEM image in Figure 2 is in line with what is anticipated for silver nanoparticles. During the nucleation and growth of silver nanocrystals from a solution or vapour phase, the atoms or ions arrange themselves to reduce the number of high-energy surface atoms, naturally leading to a globular (spherical or near-spherical) shape [20]. The globular morphology of AgNCs has several important implications for their properties and applications. Firstly, while a sphere minimizes surface area for a given volume, nanoparticles in general have a very high surface area to volume ratio, compared to their bulk counterparts. Globular nanoparticles offer a significant surface area for interactions with their surroundings, which is crucial for applications like catalysis, sensing, and antimicrobial activity. Secondly, the catalytic activity of AgNCs is influenced by the number of active sites available on their surface, which is related to their surface area and the specific crystal facets exposed. Thirdly, silver nanoparticles are well-known for their antimicrobial properties, and the high surface area of globular nanoparticles facilitates these interactions. Finally, the collective oscillation of silver nanoparticles’ conduction electrons in response to incident light gives them distinctive optical characteristics. The size and shape of the nanoparticles have a significant impact on this phenomenon, which is called surface plasmon resonance (SPR).

Globular silver nanoparticles typically exhibit a single SPR band in the visible region of the electromagnetic spectrum, in contrast with non-spherical shapes, which can have multiple SPR bands at different wavelengths, leading to different colours and optical properties that are advantageous for certain applications like biosensing. It is, therefore, often necessary to employ various synthesis strategies and capping agents to control the morphology of AgNCs and tailor their properties for specific applications like biosensing [21].

The FE-SEM image in Figure 2b shows a mostly spherical and smaller sized morphology. But some larger and elongated (rod-like or capsule-like) shapes were also observed, with dimensions measuring between 227 nm and 471 nm in width, and between 770 nm and 1.64 µm in length (average 1.21 µm). Energy dispersion X-ray spectroscopy (EDS) was used to determine the elemental composition of the AgNCs, as illustrated in Figure 2c. The EDS analysis confirms that silver is the predominant element detected. The morphology of the AgNCs is consistent with what is expected for silver nanoparticles. From the TEM image in Figure 2d, most of the particles appear at 1.7 nm radius, with a maximum diameter of 17 nm. Additionally, the AgNCs exhibited a quasi-spherical-shaped internal structure, which is expected. The TEM and SAED images in Figure 2d (insert) both show lattice fringes, confirming that the particles are crystallized.

**Figure 3:**
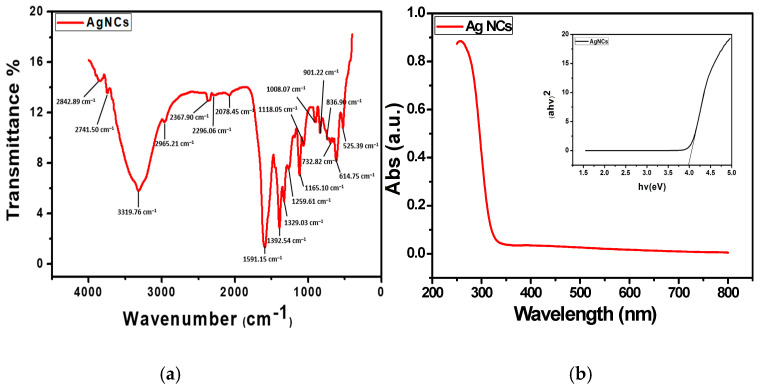
(**a**) FTIR, (**b**) UV-Vis depicting the absorption spectrum and bandgap from the Tauc plot.

The FTIR graph in Figure 3a displays noticeable peaks, which represent the distinct fingerprints of the AgNCs. Prominent peaks are seen for various stretches of bonds in the FTIR analysis. The peak at 3319.76 cm^−1^ represents the N-H stretch, 2078.45 cm-^1^ is assigned to C-H stretching vibrations, and 1591.15 cm^−1^ corresponds to the stretching vibration of the C=C bond. The peak at 1118.05 corresponds to C=O. 1392.54 cm^−1^ corresponds to C-C and C-N stretching, while 1118.05 cm-^1^ is assigned to the –C= bond, and 732.82 cm^−1^ and 614.75 cm^−1^ are for the C-H out-of-plane bend and CH bending vibrations, respectively. These peaks are comparable to unique fingerprints of silver nanomaterial, as found in the literature [22,23]. It was determined, through the utilization of UV–visible spectroscopy, that the prepared materials are nanoparticles. Figure 3b shows the plot of the absorption spectrum and Tauc’s plot obtained from the UV-Vis spectroscopic analysis of the AgNCs over a range of 200 to 800 nm. The optical band gap energy (Eg) of the AgNCs can be obtained from the Tauc plot or calculated using Tauc’s equation [24], which illustrates the relationship between the absorption coefficient and the incident photon energy of the material. Tauc’s equation is expressed as

αhν = A(hν − Eg)n(1)

where α is the absorption coefficient, hν is the photon energy, A is a constant, Eg is the optical band gap, and n is a value that varies, depending on the type of the electronic transition causing the absorption (n = 1/2 for direct transitions and n = 2 for indirect transitions). The band gap obtained from the Tauc plot for the AgNCs was 3.98 eV. This value confirms that the material exhibits good optical characteristics and will absorb in the UV-Vis range [18,25].

**Figure 4:**
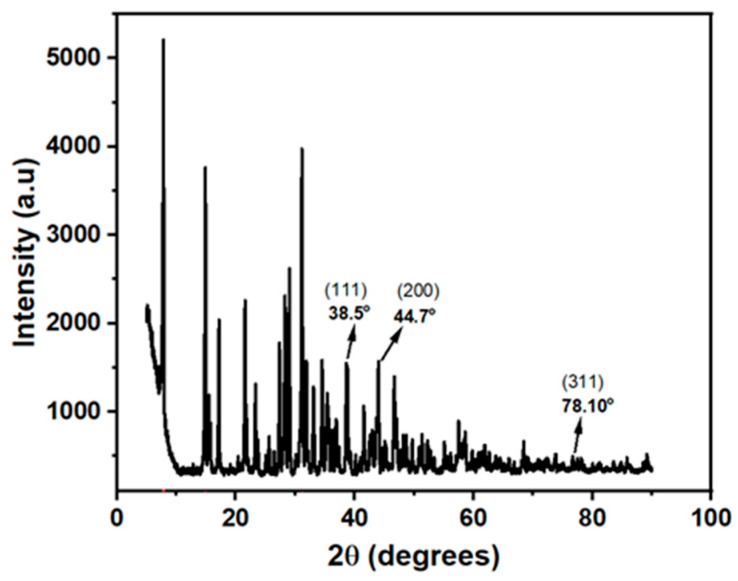
X-ray diffraction pattern.

The crystal structure of the synthesized silver nanoparticles was confirmed by using the PANanalytical XRD equipment for X-ray diffraction experiments, and the High Score program for analysis. The XRD analysis result with the diffraction pattern is shown in Figure 4.

The diffraction pattern of the powdered AgNCs shows sharp and well-defined diffraction peaks at 2θ = 38.5°, 44.7°, and 78.1°, which can be assigned to the (111), (200), and (311) reflections of the face-centred cubic (FCC) structure of pure metallic silver, respectively. This diffraction pattern is consistent with JCPDS File No 04-0783 and the literature report [26,27,28]. The produced silver nanoparticles’ outstanding crystallinity is confirmed by these distinct, strong peaks in the diffraction pattern.

**Figure 5:**
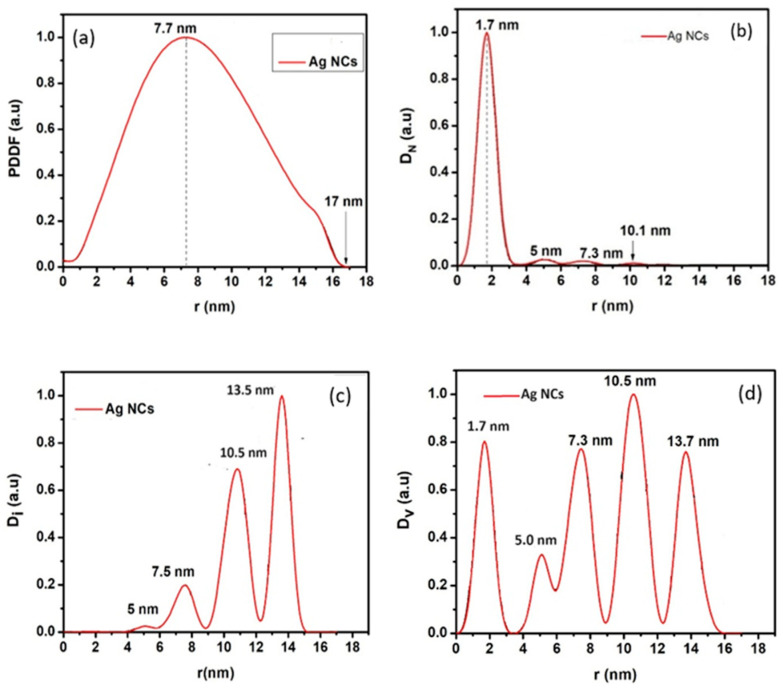
Small-angle X-ray diffraction analysis showing (**a**) internal structure, (**b**) size distribution by number, (**c**) size distribution by intensity, and (**d**) size distribution by volume.

Figure 5a–d show the small-angle X-ray scattering (SAXS) analysis which was used to probe the internal composition of the synthesized nanoparticles; they show the internal structure (a), the size distribution by number (b), the size distribution by intensity (c), and the size distribution by volume (d), respectively. As observed in these figures, most of the particles appear at 1.7 nm radius (b) with a maximum diameter of 17 nm (a). The nanoparticles showed a maximum size distribution by intensity at 13.5 nm (c), and they also exhibited a spherically shaped internal structure (a), which is expected. The large number of small particles of 2 to 8 nm is particularly advantageous in increasing the surface area of the interaction layer by adhering reactant pathogen species to the layer.

### 3.2. Analytical Application of the Immunosensor

**Figure 6:**
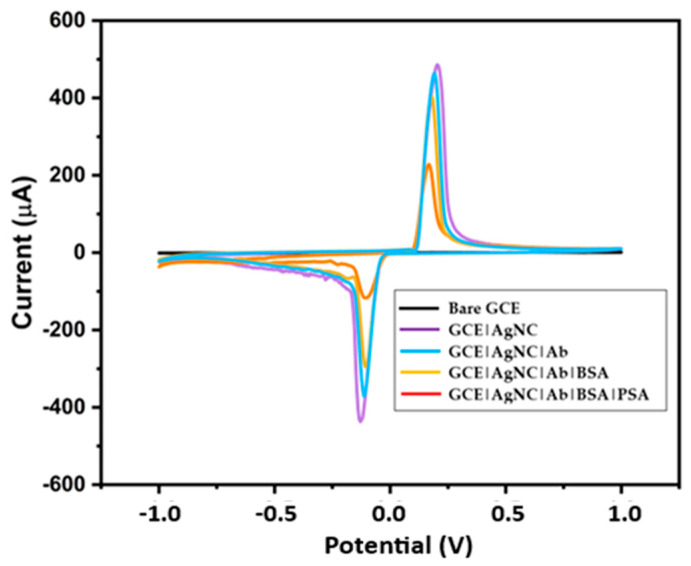
Cyclic voltammetry for the immunosensor fabrication steps.

The immunosensor development steps were characterized using CV in 10 nM PBS of pH 7.4 in the potential window −1.0 to 1.0 and at a scan rate of 50 mV/s. Figure 6 shows the CV plots of the bare GCE, GCE|AgNC, GCE|AgNC|Ab, GCE|AgNC|Ab|BSA, and GCE|AgNC|Ab|BSA|PSA. The bare GCE in Figure 6 shows no response. When AgNCs were introduced on the bare GCE, there was a significant amplification of the signal, with prominent peaks at 0.25 V, and −0.125 V, which are associated with the oxidation of Ag from Ag^0^ to Ag+ and its reduction from Ag+ back to Ag^0^. Thus, the GCE|AgNC possesses a significantly higher surface area compared to the GCE. This increased surface area allows for a greater number of antibodies to be immobilized on the electrode surface.

More immobilized antibodies lead to more binding events with the target analyte, resulting in a stronger signal, as seen with GCE|AgNC|Ab. EDC and NHS activate carboxyl groups (COOH) on the nanomaterial surface. This activation creates NHS esters that can readily react with the amine group (-NH_2_) present in the antibody molecules, forming stable covalent amide bonds. When further modified with the EDC-NHS, an observable change in signal was recorded. EDC-NHS coupling promotes a more favourable orientation of the antibody on the surface, maximizing its antigen-binding capacity and, thus, the signal. EDC-NHS chemistry enables efficient and stable immobilization of a large number of functional antibodies [29]. A slight decrease in the current response is seen in GCE|AgNC|Ab, showing that the antibody has been effectively immobilized on the surface of the electrode, thereby creating some resistance to the flow of electrons. After Ab is immobilized, there will still be uncovered areas on the electrode surface that could attract and bind other proteins or molecules present in the sample [29]. These non-specifically bound molecules can hinder the access of the target PSA antigen to the immobilized Ab, reducing the number of specific binding events and, thus, the signal. Therefore, BSA is used to block these remaining active sites on the electrode surface and effectively prevent other proteins in the sample from binding non-specifically. This guarantees that the specific binding of PSA to the Ab is mainly responsible for any subsequent rise in signal current, and not the non-specific interactions [30]. Because BSA is an insulator, it is therefore expected that the current response will diminish when it is immobilized on the electrode surface of the manufactured immunosensor, GCE|AgNC|Ab|BSA. This reaction is crucial, as it confirms that BSA, a blocking agent, has been successfully immobilized on the electrode surface. When a sample containing PSA is introduced, the PSA molecules bind non-specifically to the capture antibodies. The blocked non-specific sites ensure that only PSA binds to the sensor, reducing background noise and improving the accuracy of the detection [13]. This binding event causes a measurable change in the sensor’s properties which can alter the electrical signal, which is then measured using techniques like voltammetry. The decrease in current response observed in GCE|AgNC|Ab|BSA|PSA results from the resistance in electron transfer caused by the antibody capture of PSA for 10 ng/mL of PSA introduced in the buffer. This is because the core of the immunosensor is the specific interaction between the immobilized antibody on the sensor surface and the target antigen. Since the PSA forms a relatively large, insulating protein layer on the sensor surface, this additional biological layer increases the distance between the redox-active Ag^0^ surface and the electrolyte interface, effectively reducing the electron transfer rate to the solution-phase species. Therefore, as PSA binds to Ab on the sensor surface, this binding event modulates the properties of the sensor interface in a way that reduces the peak current, allowing for the detection and quantification of PSA, based on the magnitude of the signal decrease [29].

#### 3.2.1. Stability, Reproducibility, and Selectivity

**Figure 7:**
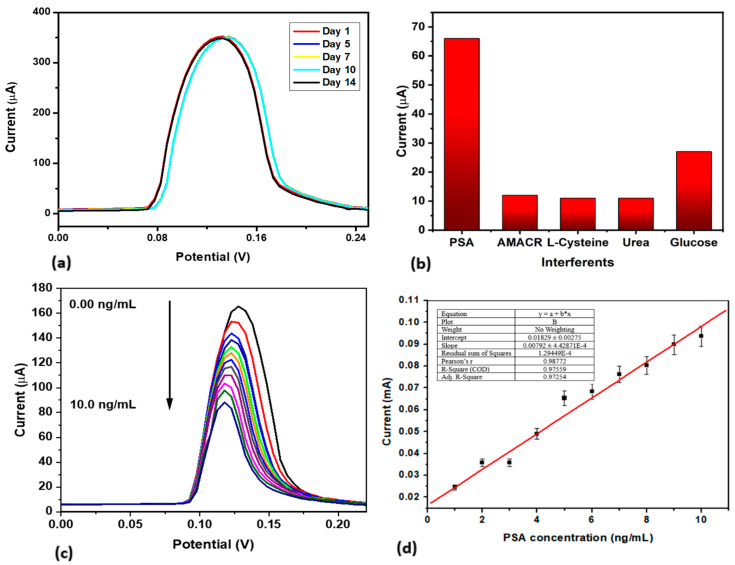
(**a**) SWV studies of the immunosensor studied over 14 days. (**b**) Interference studies of the constructed immunosensor in 10 mM PBS containing 10 ng/mL of the interferent species. (**c**) SWV of detection studies of immunosensor in 10 mM PBS at increasing concentrations of analyte from 1.0–10.0 ng/mL. (**d**) Linear calibration curve.

To study the stability of the prepared immunosensor, the current response was assessed by SWV in 10 mM PBS after one day, five days, seven days, ten days, and fourteen days of storage at 4 °C. The current response was observed as 100% after day one, 99.85% after day five, 99.42% on day seven, 99.13% on day ten, and 98.26% by day fourteen. From these results, the current response after fourteen days of storage decreased by only 1.74% (Figure 7a). This marginal current decrease confirms good stability, of 98.26%. This enhanced stability could be attributed to strong antibody–nanoparticle interactions, which may have helped minimize the degradation. To investigate the sensor’s selectivity, the interfering agents introduced were glucose, urea, L-cysteine, and alpha-methyl acyl-CoA racemase (AMACR). All these interferents are found in the blood, and were used in a 1:1 ratio to test their impact on the sensor response. The sensor GCE|AgNC|Ab|BSA|PSA was run in 10 mM PBS of pH 7.4 in the presence of 10 ng/mL of the interfering species. The current response shows that the immunosensor was selective to PSA (67%) and was not significantly affected by the interferents, whose response is between 10 to 11%, except for glucose, with a response at 27% (Figure 7b). The reproducibility of the proposed immunosensor was interrogated by measuring the current response of 10 ng/mL of PSA using four different immunosensors prepared from different electrodes under the same experimental conditions. A relative standard deviation (RSD) of 5% was calculated for the measurements, confirming that the proposed method was repeatable and analytically significant. In summary, these results show that the fabricated immunosensor produced overall good stability, reproducibility, and selectivity.

#### 3.2.2. Application in the Detection of PSA

For the detection studies, increasing concentrations of the analyte (PSA) from 1 to 10 ng/mL were added to a glass vial containing 10 mM PBS at pH 7.4 and transduced using SWV at a scan rate of 50 mV/s in the potential window of −1.0 to 1.0 V. The signal change was monitored with respect to the analyte concentrations. as shown in Figure 7c. The higher concentrations of PSA led to more binding on the sensor surface. This, in turn, resulted in a greater blockage of the electrode surface and a more significant impedance to the transfer of electrons on the surface of the sensor probe. The resultant decrease in current observed showed inverse proportionality to the concentration of PSA, which is reflected as a decrease in the overall signal response of the sensor. That is, the higher the PSA concentration, the larger the decrease in the signal compared to the baseline, due to the capture of more PSA by its antibody in CGE|AgNC|Ab|BSA|PSA, as seen in Figure 7c and the linear calibration curve in Figure 7d. Over a concentration range of 1.0 to 10.0 ng/mL, the limit of detection (LOD) was calculated to be 1.14 ng/mL, with a sensitivity of 0.0079 (±0.00044) µA/(ng/mL) and a correlation coefficient (R^2^) of 0.9877. The fabricated immunosensor in this investigation and others documented in the literature for PSA detection are compared in Table 1. Though some of the sensors reported in Table 1 have a lower LOD than that reported in this study, which can be attributed to their use of more sensitive detection techniques like DPV [31,32,33,34], and the nanomaterial composites used in their fabrication [35], which are known to increase the sensitivity of the electrochemical signals, the proposed PSA immunosensor reported in this study compares favourably to those reported in other studies in terms of linear range [18,33,35] and limit of detection [36,37]. It also exhibits promise, in that it can be used for the diagnosis of prostate cancer in its early stages within the typical range of PSA concentrations of >4.0 ng/mL [38].

The advantage of the proposed sensor developed is not only its efficacy for application in the detection of PSA, but the fact that it can be deployed in a futuristic silicon photonic-biosensor receptor layer for other biomarkers’ detection [17,18]. The proposed futuristic sensor is optical and noise-free, and can be scaled down to micro and nano dimensions. Furthermore, it can be produced as a lab-on-a-chip device for commercial scale at a low cost. This proposed design also addresses the challenge of currently available ELISA tests used in clinical settings that require expensive equipment and highly trained staff. This proposed PSA diagnostic method is simple, affordable, user-friendly, and does not need a huge investment in highly trained staff, costly equipment, or a lot of space.

**Figure 8:**
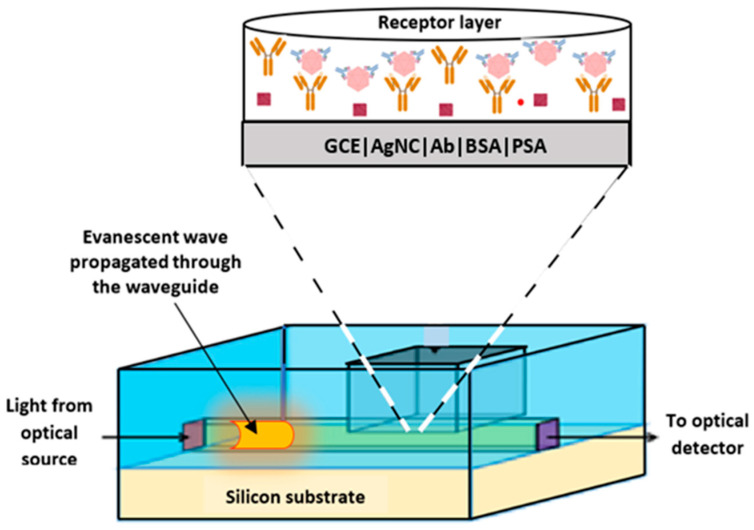
Schematic design of the proposed silicon photonic on-chip immunosensor with an optical source, evanescent-based waveguide propagation with etched bio-interaction cavity, and optical detector.

The schematic design of the proposed immunosensor is shown in Figure 8.

The sensor chip’s etched bio-interaction receptor cavity is connected to a straight silicon oxide waveguide on a silicon substrate. An evanescent wave makes up around one-third of the wave that passes through the waveguide. This wave comes from the optical source and interacts with the sample analyser in the carved cavity’s bio-interaction region. The highly sensitive receptor layer, which in this case has been modified for selective detection of PSA antigens, can provide an amplified signal of several orders of magnitude in response to the change in optical signal which is detected and processed by an optical detector, and the adjoining signal processing circuitry, all of which can be integrated on the same chip.

## 4. Conclusions

In this study, an immunosensor was developed for antigen detection using silver nanocrystals as an electrode-enhancement material. The immunosensor developed achieved a low limit of detection of 1.14 ng/mL, showed good stability and reproducibility, and was selective in the presence of interferent species. By integrating a receptor cavity interaction layer, an optical source, a waveguide, and an array of detectors, all on-chip, this proposed design aims to demonstrate the viability of a nanomaterial-enhanced silicon photonic-biosensor architecture optimized for label-free detection of PSA as a next-generation diagnostic tool for prostate cancer, with the potential to improve early detection, reduce false positives, and streamline clinical workflows.

## Figures and Tables

**Table 1:** Comparison with PSA immunosensors that have already been published.

Immunosensor/Electrode Material	Detection Method	Linear Range (ng/mL)	LOD (ng/mL)	Ref.
BSA/Anti-PSA/PTH/GQD/MG /SPE	DPV	0.0125–1.0, 1.0–80.0	0.005	[31]
AuNPs-poly(FFP-AM)-RGO/AuE	DPV	0.01–110	0.001	[32]
SPE/AuNP//Anti-body/BSA	DPV	1.0–8.0	0.55	[33]
3D-GR-Au^a^/GCE	CV, DPV	0–10	0.59	[34]
GCE/AgNP/Ab/BSA	CV	2.5–11	1.7 × 10^−1^	[18]
(MoS_2_@Cu_2_O)-Au nanocomposite	L-SAW	0.2–5	0.076	[35]
MWCNTs	EIS	0–500	1.18	[36]
Pipette-tip type biosensor	Fluorescence	0–10	1.2	[37]
GCE/AgNC/EDC-NHS/Ab/BSA	SWV	1–10	1.14	This work

## Data Availability

The original contributions presented in this study are included in the article. Further inquiries can be directed to the corresponding author.
